# Role of Occupational Health Services in Planning and Implementing of Staff COVID-19 Vaccination Clinic: A Tertiary Hospital Experience in Singapore

**DOI:** 10.3390/ijerph192114217

**Published:** 2022-10-31

**Authors:** See Ming Lim, Hwang Ching Chan, Amelia Santosa, Swee Chye Quek, Eugene Hern Choon Liu, Jyoti Somani

**Affiliations:** 1 Occupational Health Clinic, National University Hospital, Singapore 119074, Singapore; 2 Epidemiology Unit, National University Hospital, Singapore 119074, Singapore; 3 Division of Rheumatology, Department of Medicine, National University Hospital, Singapore 119074, Singapore; 4 Chairman Medical Board’s Office, National University Hospital, Singapore 119074, Singapore; 5 Yong Loo Lin School of Medicine, National University of Singapore, Singapore 119228, Singapore; 6 Department of Anaesthesia, National University Hospital, Singapore 119074, Singapore; 7 Division of Infectious Diseases, Department of Medicine, National University Hospital, Singapore 119074, Singapore

**Keywords:** COVID-19 vaccination, occupational health in hospital, vaccine hesitancy

## Abstract

Context: Healthcare workers all over the world were prioritized for vaccination against COVID-19 in view of the high-risk nature of their job scopes when vaccines were first available in late 2020. Vaccine hesitancy was an important problem to tackle in order to achieve a high vaccination rate, especially for vaccines that were developed using mRNA technology. We aimed to use the ‘3Cs’ model to address vaccine hesitancy to ensure maximal uptake of the Pfizer-BioNTech vaccine among healthcare workers in a tertiary hospital in Singapore. Methods: Various measures were used to reduce the confidence, complacency, and convenience barriers. The staff vaccination clinic was on-site and centralized, with appointments given in advance to ensure vaccine availability and to reduce wait time, providing convenience to staff. Direct and repeated communications with the staff via multiple channels were used to address vaccine safety and efficacy so as to promote confidence in the vaccines and overcome complacency barriers. To further encourage staff to get vaccinated, staff were allowed time off for vaccination when at work. Staff with a high risk of exposure to COVID-19 or those caring for immunocompromised patients were prioritized to take the vaccines first. The collection of data on adverse events was via on-site monitoring and consultation at Occupational Health Clinic (OHC). Results: Nearly 80% of staff had completed vaccination when the vaccination exercise ended at the end of March 2021. With the loosening of the contraindications to vaccination over time, staff vaccination rates reached 89.3% in early July and nearly 99.9% by the end of the year. No major or serious vaccine-related medication or administration errors were reported. No staff had anaphylaxis. Conclusions: By using the ‘3Cs’ model to plan out the vaccination exercise, it is possible to achieve a high vaccination rate coupled with effective and customized communications. This multi-disciplinary team approach can be adapted to guide vaccination efforts in various settings in future pandemics.

## 1. Introduction

On 30 January 2020, the coronavirus virus 2019 (COVID-19) was declared by the World Health Organization (WHO) as a Public Health Emergency of International Concern, and on 11 March 2020, it was designated a pandemic [1]. Since then, many countries have experienced two or even three surges in infection, with vaccination being the ultimate hope for sustained protection and a return to normalcy.

As soon as the genetic sequence of this new virus, then called novel coronavirus (nCoV) on 11 January 2020, was released [2], scientists across the world, including from Oxford University, BioNTech, and Moderna, started work immediately to develop a vaccine against this new scourge [3]. A year after COVID-19 was declared a pandemic, at least 13 different vaccines (across four platforms) are in clinical use [4], with 117 vaccines in the clinical phase and 194 vaccines in the pre-clinical phase as of 19 September 2021 [5].

Singapore was the first country in Asia to receive the BNT162b2 COVID-19 mRNA vaccine developed by Pfizer and BioNTech on 21 December 2020, and as in many countries, healthcare workers (HCWs) were prioritized to be vaccinated first by the Ministry of Health (MOH). The Pfizer-BioNTech vaccine was the first and only COVID-19 vaccine approved in Singapore then. However, given the speed at which the vaccines were produced and, in the case of mRNA vaccines, a relatively new technology to the public used for vaccine development, concerns such as vaccine safety and efficacy might lead to vaccine hesitancy.

Vaccine hesitancy refers to delay in acceptance or refusal of vaccination despite the availability of vaccination services, and addressing COVID-19 vaccine hesitancy appropriately is important to obtain high vaccination rates. The ‘3Cs’ model, namely, confidence, vaccination complacency, and vaccination convenience, was developed by the Strategic Advisory Group of Experts on Immunization, charged with advising the World Health Organization and has been used to understand the complexity of vaccine hesitancy and its determinants [6].

In the ‘3Cs’ model, confidence is defined as trust in the effectiveness and safety of the vaccines, the system that delivers them, and the motivations of the policy-makers who decide on the vaccines. Vaccination complacency is present when the perceived risks of vaccine-preventable diseases are low, and vaccination is not deemed a necessary preventive action. This is generally influenced by many factors, including health responsibilities and self-efficacy. Vaccination convenience is determined by availability, affordability, accessibility, and service delivery [6].

Concerns with regards to COVID-19 vaccines during that period revolved around vaccine safety (e.g., unknown long-term effects and safety data, and the mRNA technology used for the production of vaccines), vaccine efficacy in the long term, and suitability of vaccination for staff with pre-existing medical conditions or pregnancy/intent to conceive or breastfeed [7,8]. There were no specific guidelines on the safe set-up of COVID-19 vaccination clinics available then as well [9]. As such, we aimed to use the ‘3Cs’ model (Table 1) as a framework to address the issue of vaccine hesitancy so as to achieve high vaccination uptake and to facilitate the safe roll-out of the staff COVID-19 vaccination exercise using Pfizer-BioNTech COVID-19 vaccine on 11 January 2021 in National University Hospital, a tertiary care and academic medical center with more than 7600 staff.

## 2. Materials and Methods

### 2.1. Planning

Planning for staff COVID-19 vaccination started in mid-December 2020 by Occupational Health Clinic (OHC) in collaboration with various medical specialists and stakeholders in the hospital. OHC is an in-house clinic managing work-related health and safety issues and administering various vaccines against vaccine-preventable diseases such as varicella, measles/mumps/rubella within NUH. At the start of the COVID-19 pandemic, it had led various efforts to protect staff from contracting the infection at work, which made it most suited to lead this vaccination clinic. Guidelines from MOH regarding vaccine preparation and administration, contraindications, and side effects were followed [10], with a number of key factors to consider during this planning phase in order to ensure consistency in the vaccination exercise.

Firstly, maintaining the Pfizer-BioNTech mRNA vaccine cold chain was critical. The vial can be stored at −70 °C for 6 months and at 2–8 °C for 5 days. The vaccine is supplied in a multi-dose vial providing at least 5 doses/vial, which must be diluted just before use, stored at room temperature of 25 °C or lower, and discarded after 6 h [11]. Thus, a single centralized site for vaccination was preferred over multiple decentralized sites to facilitate control of vaccine conditions and to prevent wastage. Secondly, as there were concerns about a higher risk of severe allergic reactions with this new mRNA vaccine, we chose a site that was very near the Emergency Department (ED). Thirdly, given an expected throughput of about 500 staff per day, the vaccination center had to be spacious enough to accommodate registration, vaccination, and a mandatory 30 min post-vaccination observation period (as required by MOH), keeping to safe distancing requirements. Safe management measures included temperature taking and safe entry check-in, assigned numbered and spaced-out seats for the observation period, and wearing of surgical face masks at all times. Talking among staff was discouraged, and screening at the door identified staff who were unwell for deferment of their vaccination appointment. In line with MOH and The United States Centers for Disease Control and Prevention guidelines at the time, both vaccinators and vaccinees were required to wear surgical face masks and practice diligent hand hygiene [12].

The initial priority for appointments was given to departments with staff at higher risk of exposure to COVID-19 patients, such as staff working in intensive care units and the pandemic wards or staff caring for immunocompromised patients. Careful management of the various departments ensured that vaccination appointments were nearly fully utilized daily but spaced out. This also reduced the waiting time for staff to get vaccinated. Departments were advised to stagger vaccination among their staff to ensure business continuity if side effects such as fever occurred post-vaccination.

A dedicated group of 10 nurses in total were trained in the preparation and administration of the vaccines, recognition of signs and symptoms of adverse reactions, and treatment of anaphylaxis. This was based on the projection of 5–10 min to vaccinate a staff, with an expected throughput of about 500 staff per day. The guidelines were issued by MOH centrally, and thus, the training was standardized for all vaccinators. The number of personnel being trained was ramped up progressively based on the projected numbers of vaccinees weekly. A total of 2 administrative staff were trained to register staff as well. All the nurses and doctors from the OHC had basic cardiac life support (BCLS) certification and training in the use of adrenaline injectors. An emergency resuscitation kit with adrenaline injectors was available on-site. Emergency workflows, such as for anaphylaxis or for cardiac arrest, were rehearsed repeatedly with Emergency Department (ED) staff who would be assisting the vaccination team. Any person who had serious side effects after vaccination would be rapidly transferred to the ED for advanced life support.

Vaccine resupply runs were arranged twice weekly with MOH, with close attention to preserving the cold chain during movement and storage. Vaccine utilization and wastage were closely tracked.

### 2.2. Addressing Vaccine Hesitancy

In our hospital, it has been difficult to achieve influenza vaccine coverage above 80% among HCWs without making the influenza vaccination “mandatory” [13]. As most staff, including most doctors, were unfamiliar with mRNA vaccines prior to this COVID-19 crisis, there were concerns about “vaccine hesitancy” with the roll-out of this new type of vaccine. Even some infectious disease (ID) doctors were initially hesitant to take the mRNA vaccines due to the speed of mRNA vaccine development and the lack of long-term efficacy and safety. However, with millions of mRNA vaccinations completed in the United States of America without major incidents, it was reassuring by the time Singapore was ready to start HCW vaccination.

Clear and consistent messaging with up-to-date information was the key factor in the success of this vaccination exercise. Led by senior management, our OHC and ID doctors, human resources, and operations leaders conducted virtual town halls to inform staff and answer their questions. Staff were asked to send in questions before the town halls to better understand their concerns, which we could then address with the available objective evidence. Senior management, ID doctors, and prominent clinical leaders were invited to be vaccinated first to set an example for the staff and encourage them to get vaccinated. ID and allergy senior doctors conducted multiple education sessions, providing comprehensive updates on the following:

- The 20-year history of research and development of mRNA liposomes for protein, drug, and antigen delivery, addressing the concerns about Emergency Use Approval and the vaccines “coming out of nowhere”;
- The mechanism of action of mRNA vaccines, their efficacy data, and side effects explains the consistently high efficacy rates and safety profile in large trials;
- Explaining how vaccination was the only sustainable long-term way to protect Singapore against surges and for our staff to protect themselves, their families, and our patients;
- Helping our staff rationalize vaccination, comparing their fears of the vaccine with fears of getting infected with COVID-19. Staff were encouraged to recall their earlier great fears in March 2020 of the then little-known SARS-CoV-2 and their own earlier desire for a vaccine as the way out of the global pandemic.

There were continual updates on the vaccination status in Singapore, the cumulative number of our staff vaccinated and vaccination rates in other hospitals in Singapore, and safety profiles in countries that had vaccinated millions of people.

ID doctors also engaged departmental heads and administrative heads in smaller meetings, as well as nursing and allied health departments, tailoring updates to fill knowledge gaps and ever-increasing data on safety and efficacy so that they could help to address the concerns of their staff and nudge them to get vaccinated. In addition, whenever MOH released new vaccination guidance allowing more staff with medical conditions to be vaccinated, the heads and leads were updated to pass on the information to the unvaccinated staff. There was a consistent, clear message: vaccination, while voluntary, was important for staff and their families’ safety and for patients’ safety and should be ideally performed in “peacetime” instead of during an unforeseen surge. COVID-19 vaccination was and continues to be on a voluntary basis for our staff.

### 2.3. Staff Communications and Support Measures

Emails, department briefings, and hospital operations instructions were continuously updated and sent to all staff at regular intervals to encourage staff to sign up for their vaccination. Staff were allowed to get vaccinated during working hours, including during shifts, if the cover of duties could be arranged. They were also allowed to take medical leave for more severe post-vaccination side effects.

All staff were asked to indicate whether they wanted to be vaccinated and to sign up for vaccinations using an in-house intranet platform, the Staff Health Surveillance System. This system also had questions to screen staff for any contraindications to COVID vaccination. Departmental heads and leaders were regularly updated about the vaccination rates of their staff. This created indirect “peer pressure” among departments to improve vaccination rates and enabled them to personally discuss with and encourage their staff to get vaccinated.

OHC set up a new consult service, with walk-in, phone, or e-mail consultations for staff who had concerns about vaccination or who had had adverse experiences with other vaccines. OHC, allergy, and ID specialists worked together to address individual staff inquiries. Counseling of staff was tailored according to their needs: the emphasis could vary from clarifying doubts and protecting individuals to protecting families, colleagues, and patients.

### 2.4. Implementation

The staff vaccination clinic started at half capacity in the first week and then gradually increased to full capacity by the third week. This enabled the vaccination team to familiarize themselves with and refine good practices in vaccine handling, dilution, and tracking and to address unforeseen problems.

Throughout the exercise, no-show rates averaged 8% daily, leading to potential vaccine wastage. Staff who could not attend were encouraged to find direct replacements within their departments to take up their vaccination appointments. The clinic also started accepting walk-ins and operated a “soaker” system, calling up other staff to come and take up the unused appointments and vaccines.

A continual process of feedback resulted in the improvement of efficiency and workflows. Staff were advised to come 10 min prior to their allotted time to complete registration and to wear short-sleeved shirts, minimizing delays. The observation of giddiness and high blood pressure post-vaccination led to staff being reminded to take their chronic medications and hydrate appropriately prior to vaccination.

### 2.5. Monitoring of Adverse Effects

On-site monitoring for adverse effects for 30 min after vaccination was mandatory. If any staff had any symptoms or adverse effects, they would be monitored closely for their vital signs and assessed by an OHC doctor.

Staff were encouraged to report any adverse effects or allergic reactions to OHC after leaving the vaccination clinic via calls, emails, or walk-in consults. For potential allergic reactions or prolonged/severe side effects, they would be reviewed and reported to MOH by OHC. Together with an allergy specialist, OHC would assess and review rashes or side effects that could indicate an allergy to determine if a second dose could be administered safely.

## 3. Results

**Figure 1:**
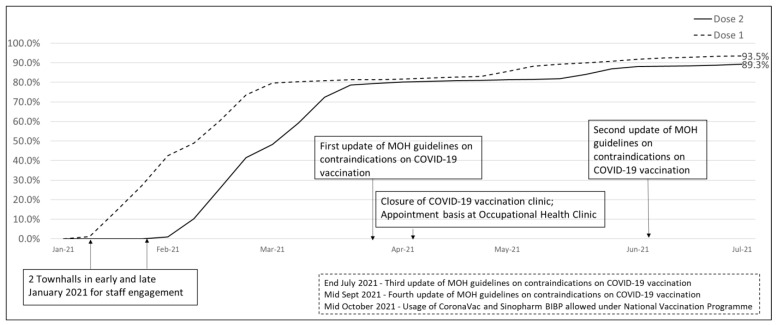
Timeline of COVID-19 vaccination exercise.

Nearly 80% (6101 out of 7671) of staff had completed both doses of the vaccine by the end of the exercise in late March 2021. Table 2 shows the demographics of the fully vaccinated staff by late March 2021. The ancillary group had the lowest vaccination rate at 75.6% among the various vocations. Staff above 40 years old generally achieved > 80% vaccination rates. With a surge in community cases in Singapore end of April 2021 and with the loosening of the contraindications to vaccination by MOH [14], staff vaccination rates reached 89.3% as of 9 July 2021, with more than 90% of staff aged 40 and above completed vaccination (Figure 1).

As of mid-May 2021, out of 1167 unvaccinated staff, 746 (63.9%) had one or more contraindications at that time, for example, medical conditions or pregnancy/breastfeeding, 351 (30.0%) preferred to be vaccinated at a later date, and 70 (6.0%) did not want to be vaccinated. As guidance on the contraindications to vaccination became less restrictive [15], the number of unvaccinated staff had dropped to 432 as of 21 July 2021. By the end of 2021, the vaccination rate among hospital staff was nearly 99.9%, with eight staff yet to be vaccinated, while the national-level vaccination rate was between 92% and 95% for various age groups ≥20 years old [16]. 

In total, 525 staff were attended to for various adverse effects by OHC during this staff vaccination exercise. The adverse effects reported were mostly mild and self-limiting such as fever, myalgia, and pain over the injection sites. No major or serious vaccine-related medication or administration errors were reported. No staff had anaphylaxis [15].

## 4. Discussion

Overall, the approach we took for our staff vaccination exercise was effective in achieving a high vaccination rate and safe, particularly considering that it was rolled out in the context of an Emergency Use Approval of a very new type of vaccine with little prior clinical experience. There were no medication or safety/screening protocol errors throughout and no wastage of vaccines. 

Fully vaccinated rates among HCWs ranged from 57.9% to 75% in other hospitals, both locally and overseas, over an estimated 3-month period [17]. Although details of how the vaccination exercises were conducted in other hospitals were not available, occupational health services in our hospital must have taken the right step in achieving high vaccination rates via a multi-disciplinary team approach based on the ‘3Cs’ model in addressing vaccine hesitancy.

The ancillary group achieved a lower vaccination rate as compared to other vocations by the end of March 2021, while medical staff achieved the highest vaccination rate of 86.1%. This was in line with another public healthcare cluster in Singapore based on the COVID-19 vaccination acceptance survey among the vocational groups [18]. The lower rate among ancillary vocation could be due to various barriers/motivations, e.g., insufficient up-to-date data to make informed decisions and not being directly involved in the care of COVID patients as compared to other vocations [7,19]. Therefore, it was essential to tailor the messaging to the needs of different groups of staff with various concerns.

Indeed, even though communication is not in the ‘3Cs’ model, it can affect vaccination uptake and cause vaccine hesitancy if poorly done so [6]. We used town halls to address the general staff populations, helping to address general concerns and questions. Engagements in the form of departmental meetings allowed more informal settings for small group sessions to answer queries. Constant communication with staff to address concerns and peer support convinced even the more hesitant staff to get vaccinated. The departmental heads were also regularly updated about the vaccination rates of their staff. For unvaccinated staff, the departmental heads would personally discuss with them to note down the reasons and refer them for further one-to-one personalized counseling at OHC if required [20]. With the updates of guidance to contraindications to vaccination, the unvaccinated staff were constantly informed via emails and departmental heads, and they could still get vaccinated within the hospital conveniently. All these addressed the Confidence and Complacency parts in the ‘3Cs’ model and played a significant role in getting hesitant staff vaccinated.

The Convenience part in the ‘3Cs’ model was adequately addressed with the well-planned and run on-site vaccination clinic with no major or serious vaccine-related medication or administration errors reported. This gained the trust of the staff, reduced barriers to vaccination, and, in turn, reduced vaccine hesitancy. We used feedback provided by staff to improve the processes and the wait time in the clinic, thus improving its efficiency. 

Certainly, having vaccinated role models among colleagues advocating for vaccination would play an important role in shifting the decision to get vaccinated [21]. In addition, positive experiences from other countries’ vaccination experience and our own staff vaccination drive’s low rate of adverse reactions were shared with staff at various platforms to reassure them that vaccination was safe, thus increasing transparency and trust, which in turn led to higher vaccination rates.

At the start and during the vaccination exercise, we considered whether to introduce positive incentives, e.g., a day off work and nominal gifts, or negative incentives, e.g., mandatory signing of declination forms through the departmental heads or directors of various departments. However, we decided against these incentives as vaccination was voluntary, and the take-up rate was satisfactory with the existing communication and support measures. We felt that vaccine incentives at a national level, for example, permission to travel without a stay-home notice post-travel, would have been more effective.

It was left to the individual departments to stagger their staff for vaccination to ensure business continuity in the event of any post-vaccination side effects resulting in staff taking sick leave. Some measures used included rostering staff to days off after vaccination and ensuring members in the same team did not take the vaccines at the same time. As for the sick leave taken weekly due to side effects reported to OHC, it ranged from around 17 to 33 staff a week (0.22% to 0.43% of total staff strength).

Although OHC had consult services that allowed concerned staff to walk in for advice, this service could have been expanded further to individual departments with low vaccine take-up rates, allowing concerns to be addressed in a non-threatening manner.

When the COVID-19 pandemic started, OHC had redeployed HCWs with medical conditions that put them at increased risk of COVID-19 complications to non-COVID-19 patient areas [22] with strict enforcement of appropriate personal protective equipment (PPE) and hand hygiene. Staff who were unvaccinated would be placed in lower-risk areas with PPE and hand hygiene to be strictly complied with.

## 5. Conclusions

By using the ‘3Cs’ model to plan out the vaccination exercise, namely confidence, vaccination complacency, and vaccination convenience, it is definitely possible to achieve a high vaccination rate coupled with effective and customized communications. Although vaccination remained voluntary, there was a strong encouragement from senior management and peers, particularly with renewed surges and emerging variants overseas. Close collaboration among OHC, nursing, pharmacy, and operations staff enabled the swift resolution of problems. These factors enabled a safe vaccination exercise with a high vaccination uptake rate, and they could be adapted as a template to guide vaccination efforts in various settings in future pandemics.

## Figures and Tables

**Table 1:** Factors for consideration in the roll-out of staff COVID-19 vaccination exercise in a tertiary hospital based on the ‘3Cs’ model.

Stages	Factors for Consideration
Planning	Confidence Early access and reference to official guidance and instructions with regard to proper vaccine storage, preparation, administration, contraindications, and side effects Quick access to emergency services Staggering of vaccination of staff within job groups to ensure business continuity Adequate manpower allocation and training (e.g., nurses and doctors) Careful management of supply chain and cold chain Complacency Prioritization of staff with a high risk of exposure to COVID-19 or those caring for immunocompromised patients Convenience Centralized vaccination site with consideration of space requirements and safe management measures Careful appointment management to ensure a high slot take-up rate
Staff Engagement Addressing Vaccine Hesitancy	Confidence Sharing of how the vaccines work, efficacy data, and side effect profiles to allay fears of new vaccines Direct and repeated communication with staff via multiple different channels, e.g., town hall meetings, emails Vaccination of senior management first to lead by example Confidence and Complacency Pitching the right messages to staff to address concerns Ease of seeking clarifications with doctors at OHC via calls, emails, or walk-in consults Regular engagement with and updates of uptake rates to departmental heads and directors to lead staff to get vaccinated Convenience Allowing time off for vaccination and utilization of hospitalization leave in the event of any side effects Usage of intranet platform, Staff Health Surveillance System, to allow easy sign-up
Implementation	Confidence Start slow to allow familiarization and development of good practices and to address unforeseen problems that can arise with increased workload Convenience Direct replacement within department is encouraged if staff is unable to turn up Have a reserve list of staff in lower priority group in the event of no-shows Explore methods to make the workflow more efficient throughout the vaccination exercise via feedback Remind staff to take chronic medications and hydrate appropriately prior in order to reduce any commonly seen post-vaccination reactions
Monitoring of Side Effects	Confidence On-site monitoring for post-vaccination side effects or allergic reactions for 30 min Posters on symptoms of anaphylaxis put up in the observation areas Attending to staff with discomfort immediately at the monitoring area Detailed recording and reporting of any side effects and allergic reactions that developed during or after post-vaccination observation by OHC and using form.gov.sg (accessed on 31 July 2022) Review of staff at OHC if they reported any potential allergic reactions or prolonged/severe side effects Reporting to MOH online in accordance with prevailing guidelines

ED: Emergency Department; MOH: Ministry of Health; OHC: Occupational Health Clinic.

**Table 2:** Demographics of fully vaccinated staff by late March 2021.

Number of Staff	Number of Fully Vaccinated Staff (%)
Total (N = 7671)	6101 (79.5)
Sex	
Male (*n* = 1439)	1224 (85.1)
Female (*n* = 6232)	4877 (78.3)
Age Group	
<20 (*n* = 8)	5 (62.5)
20–29 (*n* = 1991)	1396 (70.1)
30–39 (*n* = 2854)	2248 (78.8)
40–49 (*n* = 1499)	1308 (87.3)
50–59 (*n* = 880)	776 (88.2)
60–69 (*n* = 374)	313 (83.7)
70 and above (*n* = 65)	55 (84.6)
Vocation	
Medical/Dental (*n* = 784)	675 (86.1)
Nursing (*n* = 3240)	2621 (80.9)
Allied Health (*n* = 1567)	1221 (77.9)
Ancillary (*n* = 1380)	1043 (75.6)
Administration (*n* = 700)	541 (77.3)

## Data Availability

Not applicable.

## References

[1] World Health Organization Rolling Updates on Coronavirus Disease (COVID-19). https://www.who.int/emergencies/diseases/novel-coronavirus-2019/events-as-they-happen.

[2] Centre for Infectious Disease Research and Policy China Releases Genetic Data on New Coronavirus, Now Deadly. https://www.cidrap.umn.edu/news-perspective/2020/01/china-releases-genetic-data-new-coronavirus-now-deadly.

[3] Krammer F. (2020). SARS-CoV-2 vaccines in development. Nature.

[4] World Health Organization Coronavirus Disease (COVID-19): Vaccines. https://www.who.int/news-room/q-a-detail/coronavirus-disease-(covid-19)-vaccines?adgroupsurvey={adgroupsurvey}&gclid=CjwKCAjwwqaGBhBKEiwAMk-FtLaeP7UW7cugD86Xt5tQXYFxKqdjs-KztB9Bhx_pulG8vHfvRW3r4BoCU74QAvD_BwE.

[5] World Health Organization Draft Landscape and Tracker of COVID-19 Candidate Vaccines. https://www.who.int/publications/m/item/draft-landscape-of-covid-19-candidate-vaccines.

[6] MacDonald N.E. (2015). SAGE Working Group on Vaccine Hesitancy. Vaccine hesitancy: Definition, scope and determinants. Vaccine.

[7] Barbari A. (2021). COVID-19 Vaccine Concerns: Fact or Fiction?. Exp. Clin. Transpl..

[8] Guidry J.P., Laestadius L.I., Vraga E.K., Miller C.A., Perrin P.B., Burton C.W., Ryan M., Fuemmeler B.F., Carlyle K.E. (2021). Willingness to get the COVID-19 vaccine with and without emergency use authorization. Am. J. Infect. Control.

[9] Swift M.D., Sampathkumar P., Breeher L.E., Ting H.H., Virk A. (2021). Mayo Clinic’s Multidisciplinary Approach to Covid-19 Vaccine Allocation and Distribution. Nejm Catal. Innov. Care Deliv..

[10] Ministry of Health, Singapore COVID-19 Vaccination. https://www.moh.gov.sg/covid-19/vaccination.

[11] U.S. Food & Drug Administration Fact Sheet for Healthcare Providers Administering Vaccine (Vaccination Providers).

[12] The United States Centers for Disease Control and Prevention Interim Guidance for Routine and Influenza Immunization Services During the COVID-19 Pandemic. https://www.cdc.gov/vaccines/pandemic-guidance/index.html.

[13] Pitts S.I., Maruthur N.M., Millar K.R., Perl T.M., Segal J. (2014). A systematic review of mandatory influenza vaccination in healthcare personnel. Am. J. Prev. Med..

[14] Ministry of Health, Singapore FAQs—Safety and Efficacy of the COVID-19 Vaccine. https://www.moh.gov.sg/covid-19/vaccination/faqs---safety-and-efficacy-of-the-covid-19-vaccine.

[15] Lim S.M., Chan H.C., Santosa A., Quek S.C., Liu E.H.C., Somani J. (2021). Safety and side effect profile of Pfizer-BioNTech COVID-19 vaccination among healthcare workers: A tertiary hospital experience in Singapore. Ann. Acad. Med. Singap..

[16] Ministry of Health, Singapore Vaccination Statistics. https://www.moh.gov.sg/covid-19/vaccination/statistics.

[17] Becker’s Hospital Review Workforce COVID-19 Vaccination Rates among 8 Top US Hospitals. https://www.beckershospitalreview.com/infection-control/workforce-covid-19-vaccination-rates-among-6-top-us-hospitals.html.

[18] Tan K.W.A., Wijaya L., Lim C.T., Gan W.H. (2022). COVID-19 vaccination acceptance of healthcare workers in Singapore. Ann. Acad. Med. Singap..

[19] Dror A.A., Eisenbach N., Taiber S., Morozov N.G., Mizrachi M., Zigron A., Srouji S., Sela E. (2020). Vaccine hesitancy: The next challenge in the fight against COVID-19. Eur. J. Epidemiol..

[20] American Medical Association COVID-19 Vaccine Hesitancy: 10 Tips for Talking with Patients. https://www.ama-assn.org/delivering-care/public-health/covid-19-vaccine-hesitancy-10-tips-talking-patients.

[21] Dubov A., Phung C. (2015). Nudges or mandates? The ethics of mandatory flu vaccination. Vaccine.

[22] Hwang J., Yong E., Cheong K., Ling Z.J., Goh L.H., Lim F.S., Loh V., Bagdasarian N., Somani J., Archuleta S. (2020). Responding to the COVID-19 pandemic: The role of occupational health services in a tertiary hospital in Singapore. J. Occup. Health.

